# Virtual reality boxing: Gaze-contingent manipulation of stimulus properties using blur

**DOI:** 10.3389/fpsyg.2022.902043

**Published:** 2022-09-29

**Authors:** Annabelle Limballe, Richard Kulpa, Alexandre Vu, Maé Mavromatis, Simon J. Bennett

**Affiliations:** ^1^Inria, M2S - EA 7470, University Rennes, Rennes, France; ^2^Inria, CNRS, IRISA, University Rennes, Rennes, France; ^3^School of Sport and Exercise Sciences, Liverpool John Moores University, Liverpool, United Kingdom

**Keywords:** blur, sport, boxing, anticipation, virtual reality

## Abstract

It has been reported that behavior of experts and novices in various sporting tasks is impervious to the introduction of blur. However, studies have used diverse methods of blurring the visual stimulus (i.e., dioptric blur and Gaussian blur), and tasks that did not always preserve the normal perception-action coupling. In the current study, we developed a novel experimental protocol to examine the effect of different levels of Gaussian blur on interception performance and eye gaze data using an immersive VR task. Importantly, this provided a realistic simulation of a real-world boxing scenario (e.g., the presence of a feint prior to the onset of different combinations of punches) in which expert combat athletes (*n* = 18) experienced a first-person, adaptive viewpoint of the visual environment, which could be blurred according to their gaze location (central blur, peripheral blur, no blur). We found that participants exhibited similar interception performance in the presence of central blur or peripheral blur compared to a control condition with no blur. However, interception performance was significantly better with a central blur compared to peripheral blur. Eye gaze data indicated that although participants fixated at similar areas of interest irrespective of the presence of blur, fixation duration was significantly longer with a strong level of blur in the peripheral viewing condition than all levels of central blur and the control condition. These findings can be explained by relocating attention to different areas of the environment, which thereby influenced the perception of salient information. Participants also performed better on the first punch of a sequence preceded by a foot feint compared to arm feint or no feint. Still, irrespective of feint type, performance was significantly better on the second and third punch compared to the first punch. These findings are consistent with participants using additional information from the opponent's body movements and situational probabilities to increase performance as the sequence of punches developed. Overall, these are the first evidence for the use of VR as a means to examine gaze-contingent manipulations of the environment, and hence highlight the potential for facilitating learning and transfer to a real sporting situations.

## 1. Introduction

There have been a number of studies that have reconsidered the role of visual function in sport (Laby and Appelbaum, 2021). Rather than trying to improve performance by improving basic visual function, there has been interest in whether athletes can benefit from training in conditions that place different demands on the visual system. One such approach has been to consider whether experienced/skilled athletes can maintain, and even learn to improve their performance advantage under impoverished visual conditions. For instance, several studies have examined the effect of removing fine details by artificially blurring the visual stimulus. Jackson et al. (2009) used video recordings of a tennis serve to investigate the effect of blur, and thus the importance of detailed information (i.e., movement of specific features) compared to configural information available from movement kinematics, in groups of “poor anticipators” and “good anticipators.” The results indicated that performance of “poor anticipators” increased from the 0% blur to 20% blur video condition, whereas the “good anticipators” performance decreased. However, both groups increased their performance from the 20% to 40% blur condition. To explain the finding that blur can facilitate anticipation performance in some circumstances, the authors suggested there may have been a switch of visual strategy such that participants were more attuned to configural information in the presence of blur, and less focused on high acuity information that can be distracting.

In a subsequent study, Ryu et al. (2018) presented novice badminton players with videos that showed either normal or blurred (i.e., low-pass Gaussian filter) badminton shots. Participants were divided into three training groups: low spatial frequency (i.e., blurred video), high spatial frequency (only detailed information conserved in the video), and normal vision. The video clips showed badminton shots with different occlusion times, plus a direct performance feedback. The authors reported different effects on anticipation accuracy and reaction time when comparing the type of video training. When facing deceptive actions, the low frequency training group improved the most from pre-test to post-test, and then kept this advantage in the retention test 1 week later. This observation is consistent with the performance improvement of novices reported by Jackson et al. (2009). It seemed that the blurred video encouraged participants to focus on kinematic information and thus they were more attuned to the deceptive intent. A similar statement was made by van Biemen et al. (2018) about the perception of deceptive information by skilled football referees who trained on a decision making task in a blurred or normal vision condition. The authors found that the blurred vision group improved their response accuracy to a greater extent than the normal vision group, and specifically that the skilled referees became more sensitive to genuine fouls.

Extending the work on blurred vision but now with the aim to distinguish between covert and overt attentional strategies, and thereby the role of central and peripheral vision, Ryu et al. (2013) used a gaze-contingency display in which there was a moving window or moving mask. In the moving window condition, only a circle of 5° around the gaze fixation point was visible, whereas in the moving mask condition, central vision of 5° around the fixation point was removed from the video, leaving the participants with access to information in peripheral vision only. The authors found that expert basketball players exhibited better decision-making performance in all conditions compared to novices, thereby indicating that they possessed a better capacity to use both central and peripheral vision. To overcome the limitations of using an opaque mask (i.e., complete removal of central or peripheral vision), Ryu et al. (2015) revised their protocol by applying different levels of blur to the gaze-contingent location. The participants (18 skilled and 18 less skilled male basketball players) were required to click on a vacant court (response slide) to indicate the position of the most appropriate teammate to receive the ball, with performance measured in terms of response accuracy and response time. They found that in both peripheral blur (clear central vision) and central blur (clear peripheral vision) conditions, performance of experts was better than novices for all levels of blur. Notably, when information from a part of the visual field was unavailable (opaque central vision or opaque peripheral vision), only experts were still able to perform above the level of chance. The authors suggested that experts were able to adapt and use information based on what was available. However, novices did improve their response accuracy with moderate and high blur applied in peripheral vision (NB there was no improvement for the experts in these conditions). This positive effect is in accordance with the previous results observed on novices (Jackson et al., 2009; Mann et al., 2010b; Ryu et al., 2018).

Although offering some promising findings, one limitation of manipulating blur on a 2D video display is that it decouples perception from the motor response. Indeed, it has been reported by Mann et al. (2010b) that the effect of a blurred visual stimulus is different when participants predict ball delivery direction from a real bowler by making a verbal response only (uncoupled) or verbal response plus batting movement (coupled). In the coupled condition, performance was more resistant to the blur manipulation, such that visual clarity was not essential to anticipation performance. However, in the uncoupled condition, performance increased a little for the lowest level of blur but deteriorated for the higher levels of blur. The authors suggested that a blur manipulation encourages participants to rely more on dorsal stream processing (vision for action), and thus is of greater benefit in tasks where there is a coupling between perception and the motor response. Following this logic, it is important to recognize that the use of 2D video displays to present blurred stimuli introduces a further decoupling of perception from action by preventing access to binocular information and associated vergence eye movements. Although not an issue when blur is achieved using lenses (i.e., dioptric blur) when performing tasks in real-life settings (Mann et al., 2010b), it is not practical using these methods to introduce a gaze-contingent manipulation. Therefore, to date, it remains to be determined if the application of blur to specific locations impacts upon the performance of tasks in which there is a natural perception-action coupling that occurs in close proximity to the participant.

Here, we examined the effect of different levels of blur on interception performance and eye gaze data during an immersive virtual reality (VR) boxing task. Virtual reality offers a promising research tool as it permits the realistic simulation of real-world tasks (e.g., the presence of a feint prior to the onset of different combinations of punches thrown) with a high level of control and reproducibility (Bideau et al., 2010; Miles et al., 2012; Craig, 2013; Stone et al., 2018). Importantly, VR allows the user to interact with a first-person and adaptive viewpoint of the simulated environment in real-time (Harris et al., 2019). This preservation of the normal perception-action coupling means VR allows each individual to develop a particular interaction with the world and to experience it according to their skills, knowledge, and morphology (Gibson, 1979; Fajen and Warren, 2007). In addition, advances in technology have made it possible to record eye gaze location in the VR environment, and thus introduce and determine the impact of a gaze-contingent blur manipulation. In the current study, we expected a gaze-contingent blur to central vision would have little or no negative impact on performance because it does not disrupt the perception of motion-related information from the periphery. In fact, it is possible that blurring central vision could encourage a shift of covert attention to peripheral locations, thus facilitating the pick-up of relevant information (Ryu et al., 2015, 2016). Moreover, it could help the perception of deceptive intent by suppressing detailed information (e.g., facial expression) in the central vision thereby encouraging the use of basic kinematic information regarding movement feints. Following the above logic, we expected that a gaze-contingent blur to peripheral vision would not prevent participants from perceiving motion-related information from the periphery. However, it is possible that blurring peripheral vision might encourage a shift of attention to areas without blur such as central vision (see Ryu et al., 2015), thereby encouraging focus on less relevant or even distracting information. In this case, we can expect, the pick-up of motion-related information in the periphery to be inhibited and performance to decrease.

## 2. Materials and methods

### 2.1. Participants

Eighteen participants (5 female, 13 male) with a mean age of 29 years (± 11 years) completed the experiment. There were nine athletes who practiced mixed martial arts (French boxing, judo, qwan ki do) and nine who were expert boxers. All athletes had at least 2 years of combat sports experience. Specifically, the mixed-martial athletes had an average experience of 12 (± 7) years, and the boxers an average experience of 7.8 (± 4) years. They performed at various levels from leisure to competitive and professional events (e.g., European championship). All participants were healthy, without injuries, and had normal or corrected-to-normal vision. They signed a written consent form and were free to withdraw from the experiment whenever they wanted. The experimental protocol was made in respect of the Declaration of Helsinki and accepted by the national ethical committee (CERSTAPS IRB00012476-2021-12-05-108).

### 2.2. Task and stimuli

The experiment took place in a dark sports hall, large enough to avoid collision between the participant and the physical world. The virtual environment was built with Unity software (Version 5.6.6) and consisted of a boxing ring. The ring was of official size and participants were free to move within a space of 4 × 4 m, with a virtual opponent. The origin of the scene was placed in the middle of the ring. Motion capture of a real professional boxer was performed to create the realistic animation of the virtual boxer. Participants were immersed in the virtual environment through the use of a Head-Mounted Display (HMD, model: HTC Vive Eye Pro, Field of View: 110°, image resolution: 2,160 × 1,200 pixels) and an HTC Vive tracker attached to each wrist. Participants' hands were reconstructed in the virtual environment so that they could see them and interact naturally with the scene. Using the eye tracking system integrated in the HTC Vive Eye Pro, we recorded eye movements (1° spatial resolution, 60 Hz temporal resolution)^1^ and used the gaze location data to control in real-time the gaze-contingency manipulation. Calibration of gaze location within the virtual environment for each participant was done prior to each block, using the HTC Vive Eye pro calibration tool.

**Figure 1:**
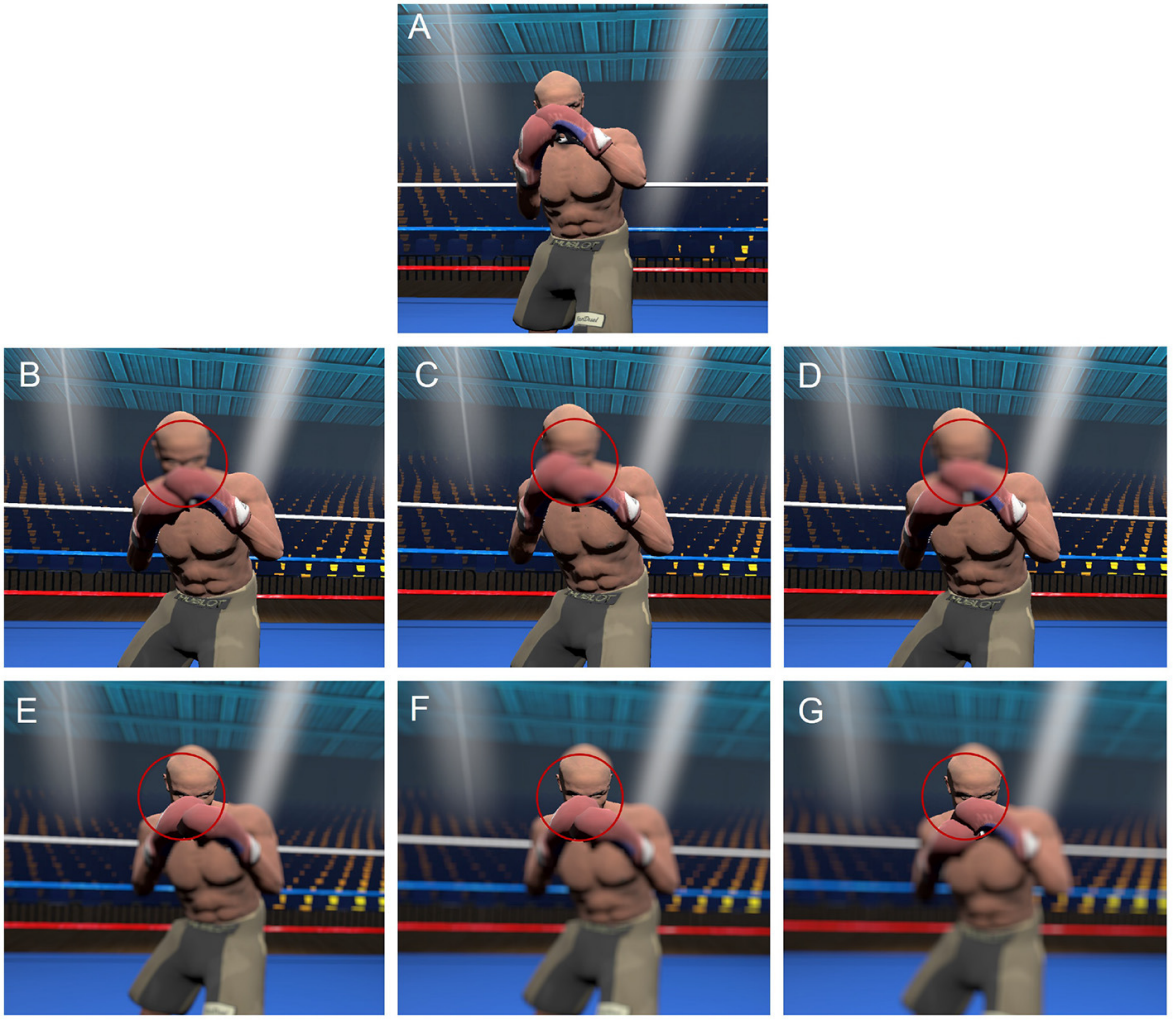
Representation of the viewing conditions. **(A)** control, **(B)** central blur level 1, **(C)** central blur level 2, **(D)** central blur level 3, **(E)** peripheral blur level 1, **(F)** peripheral blur level 2, **(G)** peripheral blur level 3. VR user view. For emphasis, we have overlaid an outline to the gaze-contingent window but this was not visible to participants.

Participants faced a virtual male boxer (see Figure 1) in the virtual boxing ring. The virtual boxer was initially shown in a guard position 1 m away from the participant and maintained a face-to-face orientation by rotating according to the participant's movement. Participants started the round (i.e., a block of trials) when they were ready by punching a red sphere located in front of them, after which the sphere disappeared. The virtual opponent then launched an attacking sequence. Participants had to block the incoming punches (with their virtual hand) by intercepting the opponent's glove during the trajectory of the attack, and thus before it touched them, with a chasing parry. Participants were instructed to only block from the start of the punch to the moment when the opponent's arm was fully extended. Auditory feedback indicated to the participant if they succeeded. No other responses, such as throwing a punch after the attack, were allowed or scored. If the participant blocked too early or too late, it was considered as a failure. Irrespective of whether the participant successfully blocked any of the incoming punches or not, the sequence continued until all three attacking punches had been thrown.

To minimize the impact of familiarity and ensure participants could not easily predict the upcoming sequence of three successive punches, we recorded 12 attacking sequences in total. These were comprised of four variations in the type and order of the three punches thrown [(i) left straight to face, right straight to face, left cross to face; (ii) left uppercut to body, right uppercut to body, left cross to face; (iii) left straight to face, left straight to face, right straight to face; (iv) left uppercut to body, left cross to face, right straight to face] by three variations in “Feint Type,” which were defined according to whether the first punch was preceded by an arm feint, foot feint, or no feint. The presence of a feint at the beginning of a sequence is commonly used by experienced boxers as a deceptive tactic to generate uncertainty regarding the onset of an attack and to create opportunities to land a successful punch. The 12 attacking sequences were presented in random order in each of the five blocks of trials, separated by a 2-min break. In each block, participants performed the virtual boxing task in one of three Viewing Conditions. In the control condition, participants were presented with normal vision of the opponent and surrounds. For the other two Viewing Conditions, a gaze-contingent blur manipulation was made relative to the point of gaze fixation (for more detail see below and Figure 1), such that participants were presented with a peripheral blur or a central blur. The control condition consisted of a single block in which there were two repetitions of the 12 sequences, resulting in 24 trials. The peripheral and central blur conditions each comprised of one repetition of the 12 sequences under three levels of blur (light, medium, and strong blur), resulting in 36 trials per block. The peripheral and central blur conditions were performed twice, such that for each level of blur, there were two repetitions of the 12 attacking sequences. See example videos of the task on central and peripheral viewing conditions in Supplementary materials.

### 2.3. Gaze-contingency manipulation

A 5° blurred circle around the gaze fixation point was used to create the central blur condition, whereas in the peripheral blur condition, the blur was only applied to stimuli outside of this 5° circle (see Figure 1). The control condition did not include any blur manipulation. A Gaussian blur, which is a 2D-convolution operator, was used to manipulate the display. It takes two inputs: the image of the participant's viewpoint generated at each frame and the Gaussian kernel whose size defines the blur intensity. A Gaussian function *G* has the following form:


(1) $$\begin{array}{l} G\left(x\right)=\frac{1}{2\pi \sigma^{2}}e^{-\frac{x^{2}}{2\sigma^{2}}} \end{array}$$

where σ is the standard deviation of the distribution, *x* is the distance from its center.

This Gaussian is applied to each pixel of the rendered image that is to be blurred. It takes into account the neighboring pixels with a weighting that is inversely proportional to its position (x,y) to the center. The degree of blurring is thus determined by the standard deviation (σ) of the Gaussian: a stronger blur is obtained with a larger convolution kernel. It is important to note that Gaussian blur does not introduce noise in the image but instead simply acts as a low pass filter that makes transitions and edges between stimuli less prominent (for more details, see Limballe et al., 2022).

The parameters of the blur were the following: light blur (σ = 100, kernel size = 13), medium (σ = 500, kernel size = 19), strong (σ = 10,000, kernel size = 25). These parameters were determined during pre-testing in which we first ascertained the strongest Blur Level that was achievable within the technological constraints (i.e., the Gaussian could be applied within the selected image refresh rate), and then decreased the kernel size and the associated sigma value, using a correspondence table. A similar approach has been used in previous work on blur (e.g., Ryu et al., 2015), where the authors used 20, 50, and 100 units blur as specified by the filters in the software used to manipulate the images.

### 2.4. Data analysis

An interception was successful when the participant stopped the opponent's incoming punch before complete extension of the opponent's arm (end of the punch). We used the collider engine from Unity to detect collisions between the participant's hand and the opponent's glove. The performance data for each punch were expressed as either a 0 for no interception or 1 for a successful interception. The collider engine was also used to identify gaze location with respect to pre-determined areas of interest (AOIs). The analysis was performed on each trial (i.e., from onset of the first punch to end of the third punch within each sequence), prior to the replacement of missing data (see below). This resulted in a percentage of the trial duration that gaze was located: (i) within AOIs associated with the opponent's body; (ii) on other areas away from the opponent; or (iii) missing due to blinks or other technical problems that prevented pupil recognition.

To reflect the global distribution of gaze throughout the entire duration of the control, peripheral blur, and central blur viewing conditions, the volume of a 3D ellipse containing 95% of the gaze data was calculated. The first stage in the process involved normalizing gaze data relative to the position of opponent's head in the 3D virtual world. Missing data (1.9% of the global observations) were then replaced using linear interpolation with the function .interpolate() from Panda Dataframe package in python. Next, the package ellipse3d from RGL (v. 0.108.3) in RStudio (v 2022.02.3) was used to draw an ellipse surrounding 95% of the gaze data. This part of the process returned a 3D ellipse mesh, which was then converted into a triangular mesh using *triangulate*_*quads* from the quadmesh (v 0.5.0) package in RStudio. Finally, the volume of the ellipse was calculated using *mesh*_*volume* from Lithics3D (v 0.4.2) package in RStudio.

To provide a more precise measure of how participants fixated eye gaze during the attacking sequences, we used a dispersion-threshold algorithm to identify instances in which there was a change in the average position (>1°) between consecutive windows (0.1 s) of eye position data (Llanes-Jurado et al., 2020). Such instances are equivalent to the start or end of a fixation. Using these data, we identified the occurrence of gaze fixations during the interval that the opponent was attacking with a punch (i.e., from the onset to the end of a punch). Having identified the start time and end time of each fixation, and hence the number of fixations, we also calculated each individual fixation duration.

### 2.5. Statistics

It was not the intention to determine if participants responded differently to the four variations in type and order of punches thrown, hence this was not considered as a factor in subsequent analysis. However, we did want to examine if participants' performance and gaze fixation data was influenced by the presence of different levels of blur in the peripheral and central conditions compared to control condition, as well as the inclusion of a feint (Feint Type). To provide a greater level of sensitivity, we also examined whether any influence of these factors was present across the first, second, and third punch (Punch Number) in an attacking sequence. Accordingly, performance and fixation data (i.e., number of fixations and mean fixation duration) were calculated from the response to eight trials (two repetitions of four variations in the type and order of punches) at each unique combination of Punch Number (1, 2, 3), Feint Type (arm, foot, none), Blur Level (1, 2, 3), and Viewing Condition (central, peripheral, control).

To analyze this partially crossed design and also take into account individual-participant variability in response to the different experimental manipulations, all data was analyzed using linear mixed effects modeling (lme4 package in RStudio, v 1.1-27.1). This is an iterative process that attempts to find the simplest model (i.e., random and fixed effects) that best fits the data. For performance and fixation data, we used a top-down strategy in which we first included all fixed effects (e.g., main and interaction terms) and a random effect of intercept and slope for Punch Number [y = Viewing condition * Feint Type * Punch Number * Blur Level + (1+ Punch Number ∣ Participant)]. We then removed fixed effects sequentially based on their statistical significance determined using Wald Chi Squared tests (CAR package in RStudio, v 3.0-12), and provisionally retained those that returned *p*-values of 0.1 or less. The reduced-effects model was compared to the full-effects model with conditional R2 (MUMIn package in RStudio, v 1.46.0) and AIC. It was also compared to a reduced model with the same fixed effects but only a random intercept. In the final reduced model, only fixed effects at *p* ≤ 0.05 were deemed meaningful and further analyzed using Bonferroni-corrected pairwise comparisons. To this end, we used the EMMEANS package (v 1.7.2) to compare estimated marginal means, and thereby take account of the partially crossed design. Performance data (number of successful trials out of eight per combination of independent variables) were modeled according to a binomial distribution with a logit link function. Number of fixations (total number of fixations across eight trials per combination of independent variables) were modeled according to a Poisson distribution with a log link function. Fixation duration (mean fixation duration across eight trials per combination of independent variables) were modeled according to a normal distribution with an identity link function.

To analyze the global measures of gaze distribution, linear mixed effects modeling with an identity link function was also used but with AOI and Viewing Condition as fixed effects for gaze location, and Viewing Condition as a fixed effect for 3D ellipse volume. Each participant was initially modeled with a random intercept and slope for Viewing Condition. The same iterative approach to removing fixed and random effects was used as described above.

## 3. Results

### 3.1. Performance data

**Figure 2:**
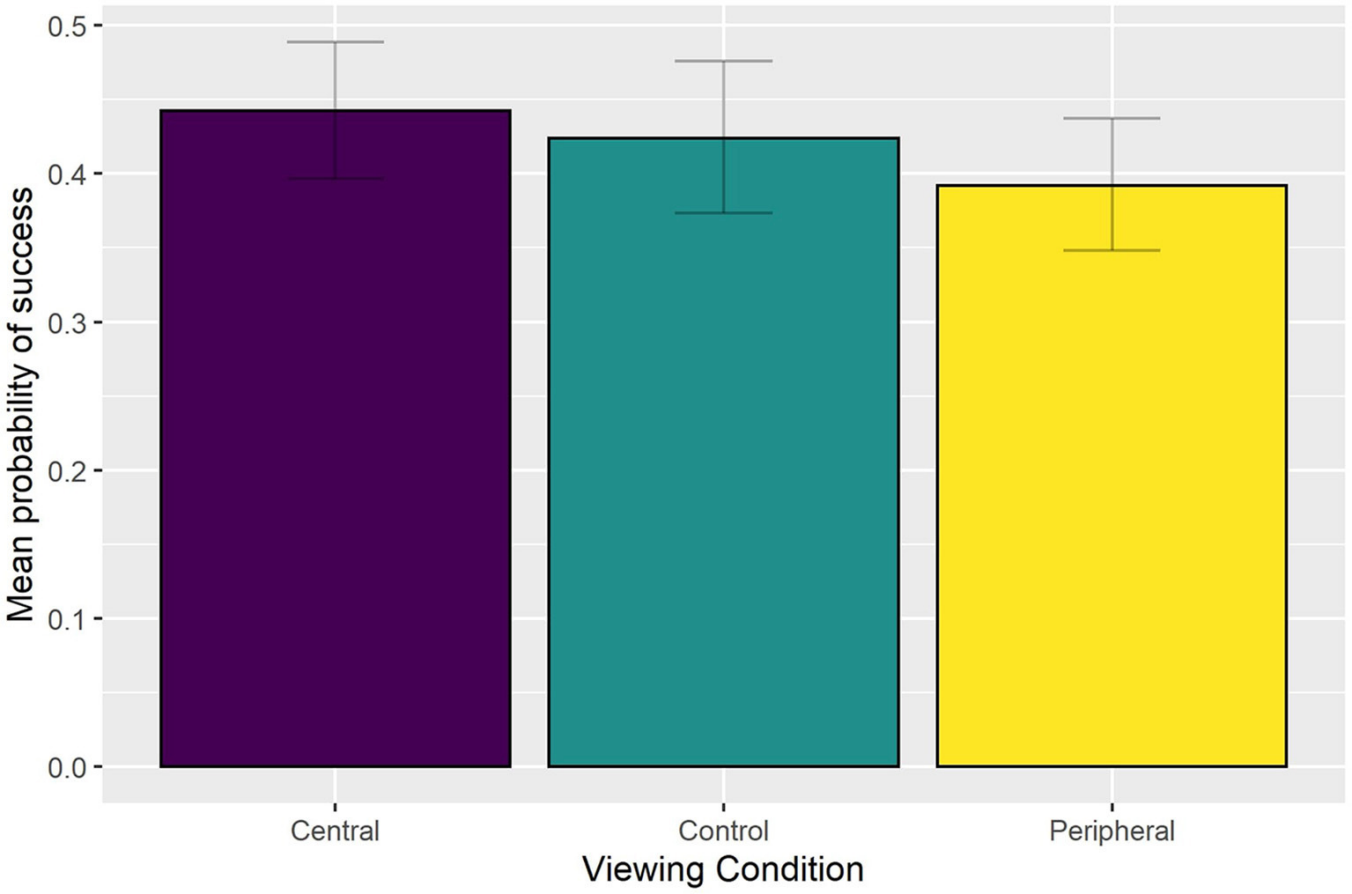
Group mean probability of a successful interception as a function of Viewing Condition.

Having removed non-significant effects found in the full model (AIC = 3,835; conditional *R*^2^ = 0.56), the final reduced model (AIC = 3,770, conditional *R*^2^ = 0.55) indicated a main effect of Punch Number [$\chi_{\left(2\right)}^{2}$ = 138.545, *p* < 0.0001], Viewing Condition [$\chi_{\left(2\right)}^{2}$ = 18.767, *p* < 0.001], and Feint Type [$\chi_{\left(2\right)}^{2}$ = 46.830, *p* < 0.001], as well as significant Punch Number by Feint Type interaction [$\chi_{\left(4\right)}^{2}$ = 63.872, *p* < 0.001]. Bonferroni-corrected pairwise comparisons indicated that performance in the central blur condition (0.442) was significantly (*p* < 0.001) better than the peripheral blur condition (0.392) but not the control condition (0.424) (see Figure 2).

**Figure 3:**
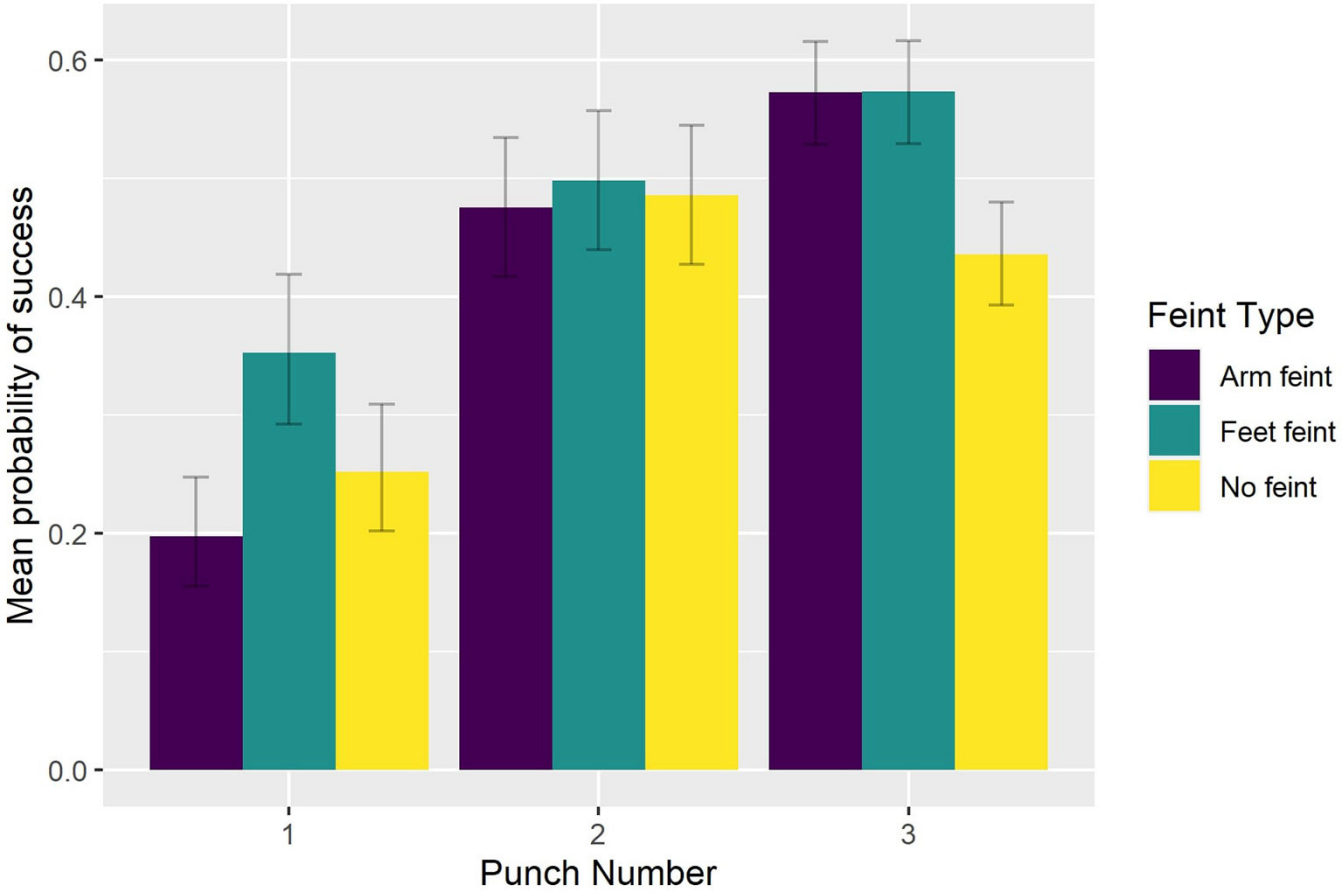
Group mean probability of a successful interception as a function of Feint Type and Punch Number.

As can be seen in Figure 3, participants intercepted significantly more of the first punches in a sequence when they were preceded by a foot feint (0.353) than arm feint (0.197, *p* < 0.0001) or no feint (0.252, *p* < 0.0001). Still, their performance was significantly better on the second and third punch compared to the first punch within a sequence irrespective of whether a feint was present or not (*p* < 0.0001).

### 3.2. Eye gaze data

At a global level (i.e., across the entire block duration), a reduced model with a fixed effect of Viewing Condition [$\chi_{\left(2\right)}^{2}$ = 26.931, *p* < 0.0001] and random intercept (AIC = 32.749; conditional *R*^2^ = 0.87) indicated a significantly better fit [$\chi_{\left(2\right)}^{2}$ = 21.85, *p* < 0.0001] of the 3D ellipse volume data than an intercept only model (AIC = 768; conditional *R*^2^ = 0.76). Bonferroni-corrected pairwise comparisons indicated that the 3D ellipse volume was smaller in the control condition (43.1 cm^3^) compared to the peripheral blur (67.9 cm^3^) and central blur (68.6 cm^3^) conditions (*p* < 0.0001).

**Figure 4:**
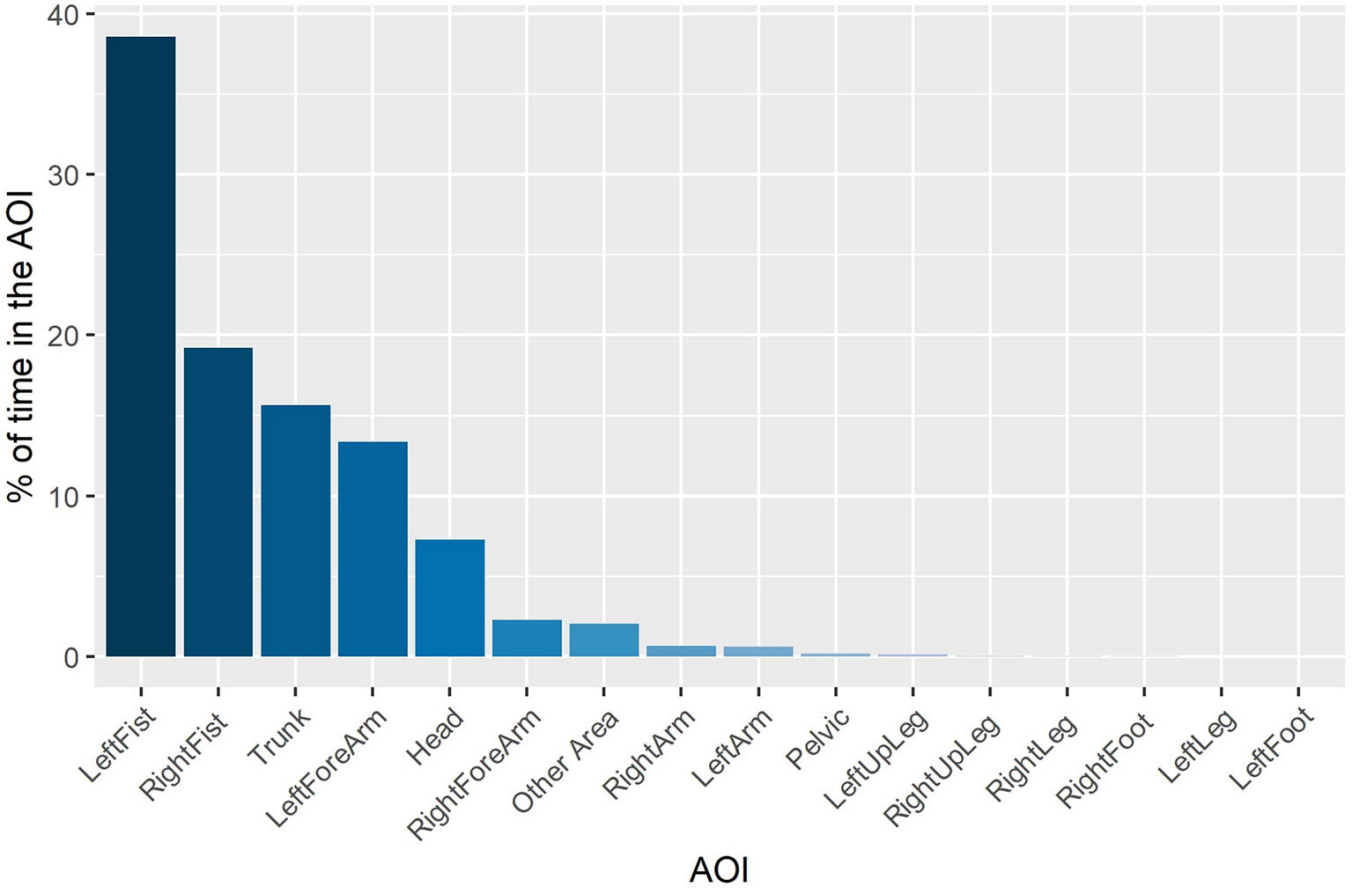
Percentage of time that gaze was located in the AOIs.

The finding of a relatively small volume that included 95% of eye gaze data across the entire duration of each viewing condition was confirmed by analysis of gaze location with respect to the AOIs within each trial. The full effects model (AIC = 7598.499; conditional *R*^2^ = 0.803) did not differ from the reduced model (AIC = 7616.348, conditional *R*^2^ = 0.806) in which there was a significant main effect of AOI [$\chi_{\left(15\right)}^{2}$ = 5134.5, *p* < 0.0001]. Observation of the estimated marginal means revealed that 95% of eye gaze within each trial was located within 6 of the 16 AOIs, which were mostly distributed on the Head, Tunk, and distal ends of the two arms (see Figure 4).

### 3.3. Fixation data

#### 3.3.1. Number of fixations

**Figure 5:**
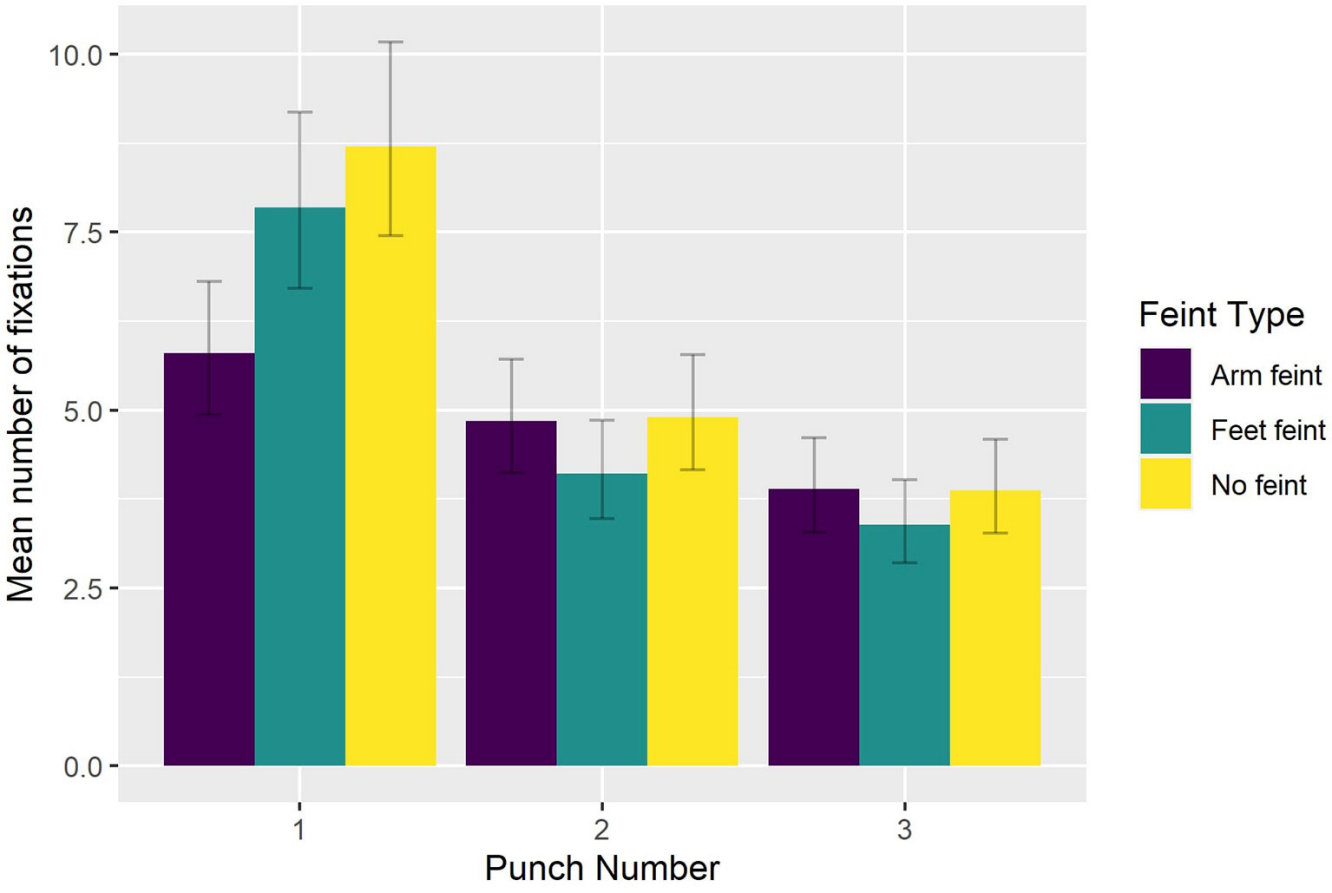
Group mean number of fixations as a function of Feint Type and Punch Number.

Having removed non-significant effects found in the full model (AIC = 4,743; conditional *R*^2^ = 0.50), the reduced model (AIC = 4,665, conditional *R*^2^ = 0.49) returned significant main effects of Punch Number [$\chi_{\left(2\right)}^{2}$ = 549.755, *p* < 0.0001] and Feint Type [$\chi_{\left(2\right)}^{2}$ = 35.117, *p* < 0.0001], and a significant interaction between Punch Number and Feint Type [$\chi_{\left(4\right)}^{2}$ = 60.066, *p* < 0.001]. Bonferroni-corrected pairwise comparisons indicated that the number of fixations (see Figure 5) decreased from the first to second punch (*p* < 0.03), and then again from the second to third punch (*p* < 0.007) for sequences that began with either no feint (8.70, 4.90, 3.88) or an arm feint (5.79, 4.85, 3.89). For sequences that began with a foot feint, there was a reduction (*p* < 0.0001) in the number of fixations from the first (7.85) to second punch (4.10), followed by a smaller decrease (*p* = 0.09) from the second to third punch (3.39).

#### 3.3.2. Fixation duration

**Figure 6:**
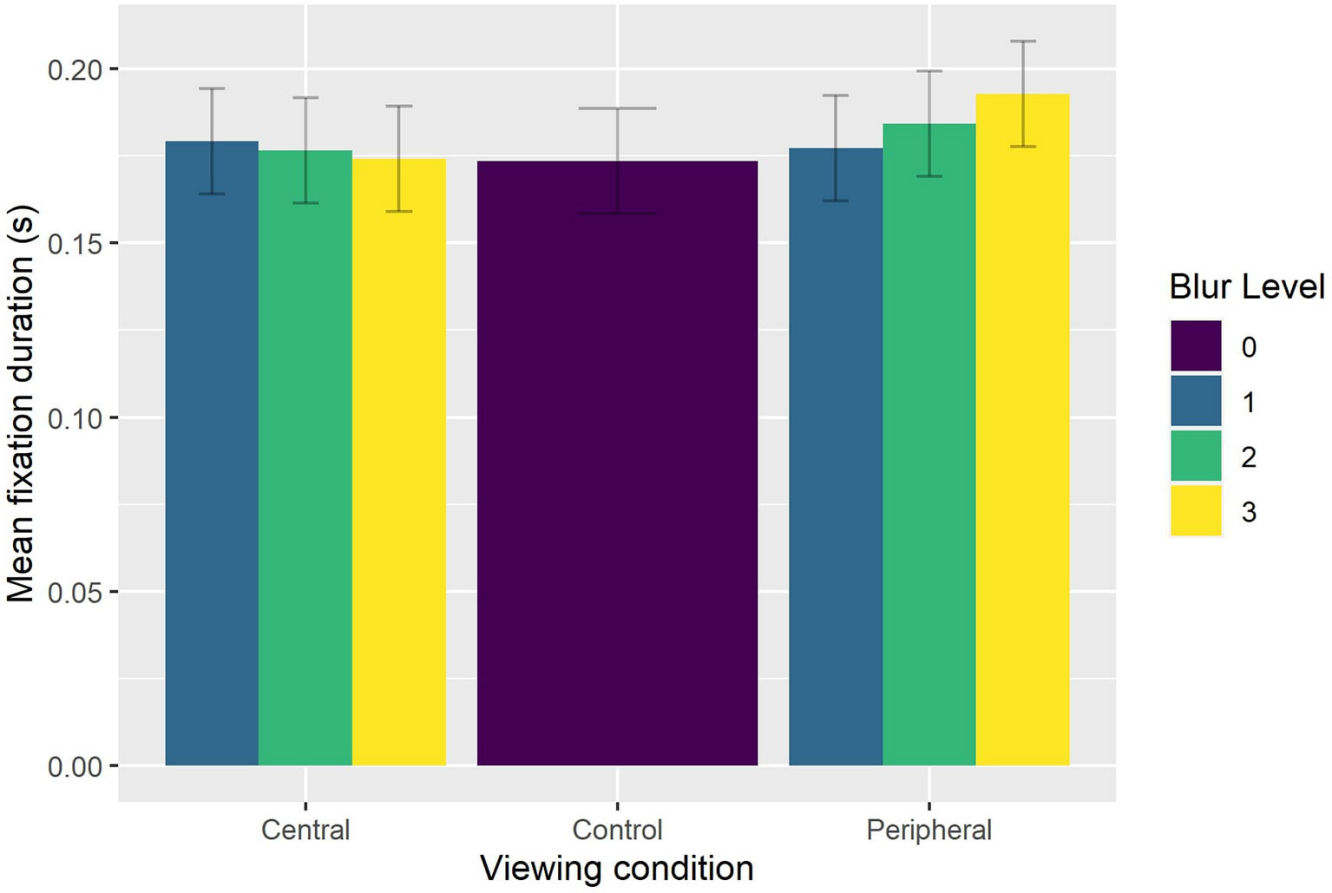
Group mean fixation duration as a function of Viewing Condition and Blur Level.

**Figure 7:**
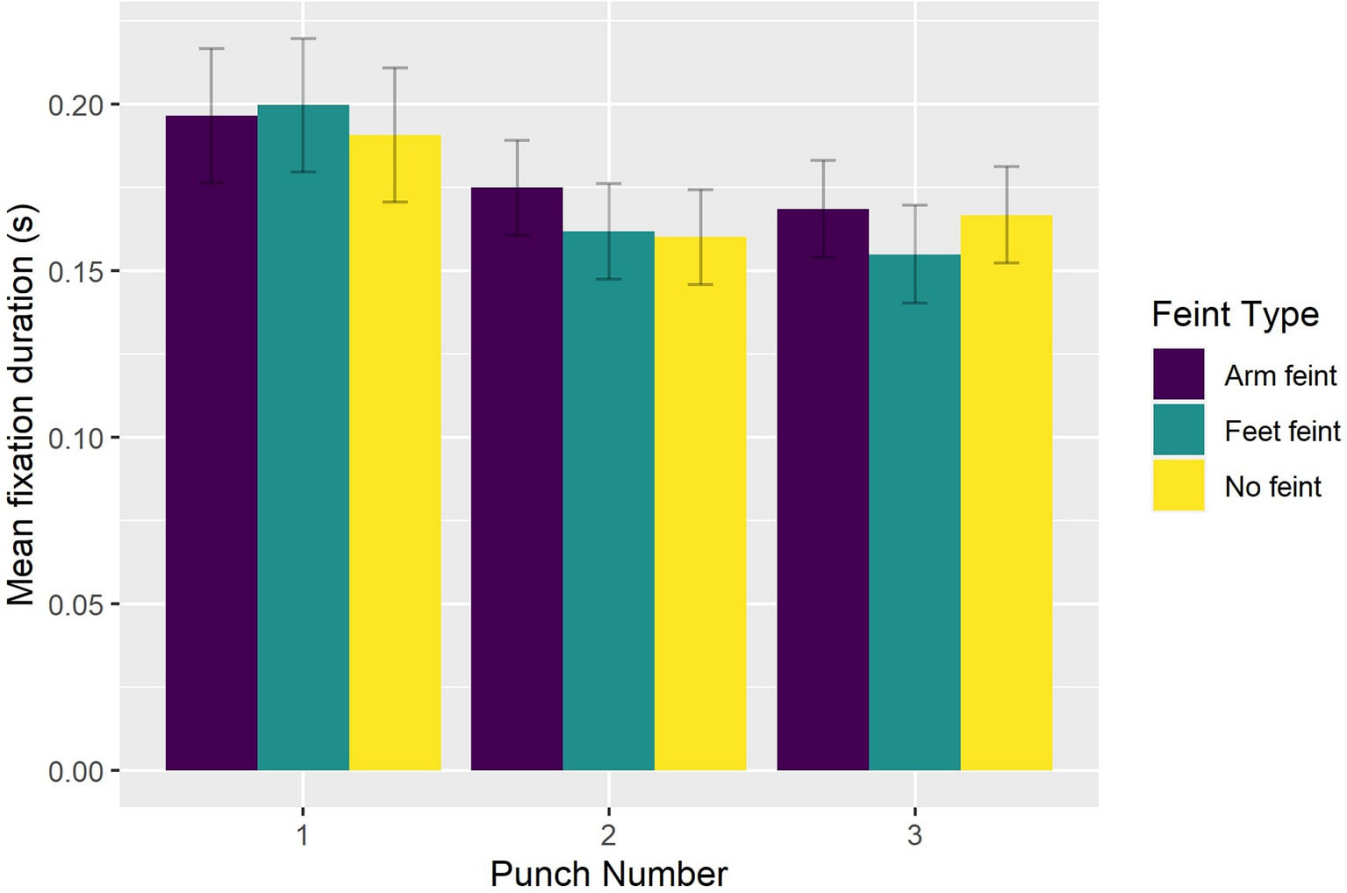
Group mean fixation duration as a function of Feint Type and Punch Number.

The reduced model (AIC = -3,892, conditional *R*^2^ = 0.47) indicated significant main effects of Viewing Condition [$\chi_{\left(2\right)}^{2}$ = 19.822, *p* < 0.0001], Feint Type [$\chi_{\left(2\right)}^{2}$ = 10.356, *p* < 0.006], and Punch Number [$\chi_{\left(2\right)}^{2}$ = 28.872, *p* < 0.0001], which were superseded by significant interaction effects between Viewing Condition and Blur Level [$\chi_{\left(4\right)}^{2}$ = 10.815, *p* < 0.025] and Punch Number and Feint Type [$\chi_{\left(4\right)}^{2}$ = 14.784, *p* < 0.006]. Bonferroni-corrected pairwise comparisons indicated that fixation duration was significantly (*p* < 0.035) longer in the peripheral viewing condition when faced with the strong level of blur (0.189 s) compared to the light level of blur (0.176 s). Fixation duration in the central viewing condition did not differ between the light (0.174 s), medium (0.172 s), or strong (0.172 s) levels of blur, or compared to the control condition (0.170 s). However, fixation duration in the central viewing condition and control condition was shorter (all *p* < 0.004) than that exhibited with the strong level of blur in the peripheral viewing condition (see Figure 6). Finally, fixation duration was significantly longer (all *p* < 0.002) on the first punch compared to the second punch for sequences that began with either no feint (0.191 vs. 0.175 s) or a foot feint (0.200 vs. 0.162 s). There was no decrease in fixation duration from the second to third punch irrespective of Feint Type (see Figure 7).

## 4. Discussion

There have been a number of studies indicating that visual and visual-motor behavior does not deteriorate (Mann et al., 2010a; Ryu et al., 2018; van Biemen et al., 2018), or can even be improved (Jackson et al., 2009; van Biemen et al., 2018), when the visual stimulus is artificially blurred. However, as was recently noted by Limballe et al. (2022), previous studies have included diverse approaches to blurring the visual stimulus (i.e., dioptric blur and Gaussian blur achieved using different methods), different populations and sporting tasks, as well as varied experimental manipulations that do not always preserve the normal perception-action coupling. Therefore, to improve upon the methodological limitations of previous work, in the current study we used an immersive VR boxing environment viewed through an HMD with an integrated eye tracker to examine if interception performance and eye gaze data of expert combat athletes are mediated by different levels of blur in the central or peripheral visual field, and whether this is influenced by the presence of deceptive information at the onset of an attack sequence.

In line with the finding that performance of expert basketball players (Ryu et al., 2015), badminton players (Ryu et al., 2018), cricketers (Mann et al., 2010a), and football referees (van Biemen et al., 2018) is relatively impervious to the presence of blur, we found that interception performance of expert combat athletes did not deteriorate significantly following a gaze-contingent blur manipulation in our virtual boxing environment. In fact, interception performance did not differ significantly between the central blur condition (0.442) and the control condition (0.424) or between the peripheral blur condition (0.392) and the control condition. The only significant difference was between the central blur and peripheral blur conditions. Moreover, we found that there was no main effect of Blur Level or interaction between Viewing Condition and Blur Level. The implication, therefore, is that the inferior interception performance in the peripheral blur condition compared to the central blur condition was evident at the lowest level of blur, and that even the highest level of blur in central vision did not impact negatively upon performance. Such findings may at first seem difficult to reconcile with reports that expert athletes make greater use of motion information available in peripheral vision (Vater et al., 2019; Williams and Jackson, 2019), which is more sensitive to low-spatial, high-temporal frequency visual stimuli, and thus should be less sensitive to the application of blur. However, as pointed out by Ryu et al. (2015), there are reports that blur causes participants to shift attention toward areas of the visual field that are not blurred (Enns and MacDonald, 2013), which they suggested in their study encouraged novice participants to attend to more salient information in central vision. Following the same logic, it is possible in the peripheral blur condition of the current study that expert combat athletes' attention was drawn to the window of clear central vision. By doing so, participants may have been less aware of relevant information available from more peripheral locations and potentially more focussed on high acuity details such as facial expression, which can act as a distractor in combat sports (Petri et al., 2019). Therefore, even though expert combat athletes may have been able to perceive and extract relevant information from peripheral vision in the presence of blur per se, they were less attentive to this area of the display and their performance deteriorated compared to the central blur condition.^2^ A similar attentional mechanism could have been at play in the central blur condition, where it is possible that participants were less focussed on distracting, high acuity details, and instead attended to motion information about the attacking sequence of punches. Whether the presence of a gaze-contingent central blur in the current study encouraged expert combat athletes to focus on a reduced amount of information and/or the more salient information given their level of expertise remains to be determined.

Interestingly, the presence of blur in the current study did not impact upon participants' ability to intercept significantly more of the first punches in a sequence when they were preceded by a foot feint (0.353) than arm feint (0.197), or no feint (0.252). The facilitative effect of a foot feint was present in all Viewing Conditions but was no longer present for the second and third punch in the sequence. As might be expected given expert combat athletes' knowledge of how an attacking sequence would likely develop, interception performance was significantly better on the second and third punch compared to the first punch within a sequence irrespective of whether a feint was present or not. Why, then, if the peripheral and central blur conditions encouraged participants to shift attention to different areas of the display (e.g., to the window of clear central vision in peripheral blur), did they exhibit a similar benefit from the presence of a foot feint preceding the first punch? To answer this question, it is relevant to note that unlike a real boxing match, where a feint is used by the attacking boxer to deceive the opponent, in the current study the feints were always followed by one of four pre-determined sequences. Thus, although participants would not have known which type of punch (e.g., straight left, straight right) to expect following a foot feint, they would have known that the feint was followed by an attacking sequence. Accordingly, it can be expected that simply becoming aware of the foot feint movement provided participants with an additional cue to the sequence onset as it clearly showed a forward movement of the opponent, and that this was still possible in the presence of a peripheral blur. Conversely, an arm feint was not associated with a clear forward body movement and thus did not provide an unambiguous cue concerning sequence onset.

To better understand how participants oriented overt visual attention in the current study, we quantified how eye gaze was located across the entire block duration (i.e., including intervals between attacking sequences). We found that eye gaze was located within a relatively small 3D ellipse volume data in the control condition (43.1 cm^3^), which increased significantly in the presence of a peripheral blur (67.9 cm^3^) and central blur (68.6 cm^3^). However, the effect of Viewing Condition was not evident when eye gaze data across the entire block duration was expressed with respect to AOIs. Of 16 possible AOIs, 95% of eye gaze data was located on the Head, Trunk, and distal ends of the two arms. This confirms previous findings regarding the relatively concentrated location of overt attention to the upper body and head in French boxers (Ripoll et al., 1995) and karateka (Williams and Elliott, 1999). To determine more precisely how participants fixated eye gaze, we next analyzed the number and duration of fixations during only the three punches of attacking sequences. For the number of fixations, there was a decrease from the first to second punch, and then again from the second to third punch for all punch sequences, although the effect was less pronounced for punch sequences that began with a foot feint. There was also a decrease in fixation duration from the first punch compared to the second and third punch in sequences that began with no feint or a foot feint. As suggested above, this different pattern of fixations on the first compared to second and third punch could have resulted from expert combat athletes being better able to anticipate an attacking sequence once it had begun. Despite there being no difference in number of fixations *per se*, fixation duration was longer in the peripheral viewing condition when faced with the strong compared to the light level of blur. No effect of blur level on fixation duration was evident in the central viewing condition, which resulted in similar fixation duration as the control condition. Subsidiary classification of fixations according to their individual duration (50–150 ms—ambient fixation; >250 ms—focal fixation, see Negi and Mitra, 2020) indicated that participants exhibited fewer ambient and more focal fixations in the peripheral blur conditions (i.e., 1,204 and 458), compared to central blur conditions (i.e., 1,373 and 436). In addition, there were more ambient and fewer focal fixations (i.e., 423 and 136) in the light peripheral blur condition, compared to the strong peripheral blur condition (374 and 178). Based on the suggestion that focal fixations reflect the conscious perception of objects in central vision, it follows that the increase when faced with a strong level of blur in the peripheral viewing condition could be associated with the proposed shift toward processing high acuity, distracting information from the opponent's facial expression (Petri et al., 2019).

The notion that focal fixations and ambient fixations are processed through different but complimentary neural pathways (e.g., ventral and dorsal; see Pannasch and Velichkovsky, 2009), provides an interesting avenue for future research on the impact of refractive blur in sport. Indeed, it was previously suggested by Mann et al. (2010a) that the resilience of interception performance to refractive blur (i.e., to the entire visual field) could be explained by a reliance on visual information processed in the dorsal pathway, which is sensitive to visual-spatial motion and thus less affected by a reduction in visual acuity. In future work, it will be relevant to conduct more detailed analysis of eye movements in dynamic sports task to better understand the distribution of ambient and focal fixations as a proxy to underlying neural processing. For example, while we were able to determine fixation number and duration using a dispersion-threshold algorithm, there were limitations in the spatial (1°) and temporal (60 Hz) resolution of the eye tracking technology within the HMD. This prevented us from precisely quantifying saccadic eye movements, and particularly those with small amplitude (e.g., < 2 − 3°), which tend to precede focal fixations. As eye tracking technology embedded within HMDs improves, it will be possible to determine more precisely how eye gaze is moved between AOIs (e.g., use of a visual pivot; Vater et al., 2019) and thus the underlying processes associated with improved performance.

In an effort to control and standardize the parameters of the VR task in the current study, we chose to evaluate participants' performance based on the effectiveness of intercepting three punches within an attacking sequence. This required the use of a chased parry, which in a combat situation is not commonly used by experts. More typically, an expert boxer will try to avoid being hit and will therefore favor partial or total dodging rather than defending with the hands (Davis et al., 2015), such as with a chased parry. Moreover, in English boxing, expert behavior consists of systematically leaving the opponent's punching axis and not staying within his punching distance. In our virtual task, the attacking opponent did not move back and forth, but instead pivoted to keep their punch axis oriented toward the participant. As a result, the participant had to adjust their distance from the opponent in order to create a punching distance that enabled them to achieve an interception of the opponent's attacking punch. To provide a standardized performance evaluation, we took the number of intercepted punches in the sequence into account. However, in combat, a boxer can voluntarily accept to receive one or two blows, can counterattack from the first or second blow of the sequence, or can go out of the axis as quickly as possible. In combination, then, our effort to maintain internal validity may have reduced the realistic nature of the combat scenario for experts. Although probably suitable for novices, in future work with experts it could be relevant to modify the virtual boxing task such that the attacking opponent only delivers a single punch instead of a sequence but requiring a more complex and typical response (e.g., avoid the blow and counterattack).

## 5. Conclusion

Combined, these data from an immersive and adaptive first-person VR setting contribute to our understanding of how gaze-contingent blur influences interception performance and eye gaze of combat sport athletes when faced with a sequence of attacking punches. Moreover, these are the first findings to highlight the potential for using VR to provide a controlled manipulation of the visual environment while at the same time preserving a normal perception-action coupling. In future work, it will be relevant to consider whether VR training with a gaze-contingent blur can facilitate learning and transfer to a real boxing situation.

## Data availability statement

The raw data supporting the conclusions of this article will be made available by the authors, without undue reservation.

## Ethics statement

The studies involving human participants were reviewed and approved by CERSTAPS IRB00012476-2021-12-05-108. The patients/participants provided their written informed consent to participate in this study.

## Author contributions

AL, RK, and SB explored literature, wrote the original draft, review, and edited the final manuscript. AL and AV designed the VR environment. AL and MV implemented the blur. AL, SB, and AV explored and processed the data and statistics. All authors approved the final version of this manuscript.

## Conflict of interest

The authors declare that the research was conducted in the absence of any commercial or financial relationships that could be construed as a potential conflict of interest.

## Publisher's note

All claims expressed in this article are solely those of the authors and do not necessarily represent those of their affiliated organizations, or those of the publisher, the editors and the reviewers. Any product that may be evaluated in this article, or claim that may be made by its manufacturer, is not guaranteed or endorsed by the publisher.

## Footnotes

^1^The native temporal resolution of the eye tracker was 90 Hz, but it was only possible to record eye gaze at 60 Hz due to processing limitations when rendering the immersive 3D virtual environment.

^2^Also, it is possible that the application of a Gaussian blur in peripheral vision could have disturbed participants' spatial orientation in the ring. For example, although the presence of blur is a complementary depth cue (Held et al., 2012; Langer and Siciliano, 2015), we did not apply a blur gradient that changed as a function of depth or in accord with the physiological optics of the retina (see Limballe et al., 2022).

## References

[B1] Bideau B. Kulpa R. Vignais N. Brault S. Multon F. Craig C. (2010). Using virtual reality to analyze sports performance. IEEE Comput. Graph. Appl. 30, 14–21. 10.1109/MCG.2009.134 20650707

[B2] Craig C. (2013). Understanding perception and action in sport: how can virtual reality technology help? Sports Technol. 6, 161–169. 10.1080/19346182.2013.855224

[B3] Davis P. Benson P. R. Pitty J. D. Connorton A. J. Waldock R. (2015). The activity profile of elite male amateur boxing. Int. J. Sports Physiol. Perform. 10, 53–57. 10.1123/ijspp.2013-0474 24912199

[B4] Enns J. T. MacDonald S. C. (2013). The role of clarity and blur in guiding visual attention in photographs. J. Exp. Psychol. Hum. Percept. Perform. 39, 568–578. 10.1037/a0029877 22984991

[B5] Fajen B. R. Warren W. H. (2007). Behavioral dynamics of intercepting a moving target. Exp. Brain Res. 180, 303–319. 10.1007/s00221-007-0859-6 17273872

[B6] Gibson J. (1979). The Ecological Approach to Perception. Boston, MA: Haughton Mifflin.

[B7] Harris D. J. Buckingham G. Wilson M. R. Vine S. J. (2019). Virtually the same? How impaired sensory information in virtual reality may disrupt vision for action. Exp. Brain Res. 237, 2761–2766. 10.1007/s00221-019-05642-8 31485708 PMC6794235

[B8] Held R. T. Cooper E. A. Banks M. S. (2012). Blur and disparity are complementary cues to depth. Curr. Biol. 22, 426–431. 10.1016/j.cub.2012.01.033 22326024 PMC3298574

[B9] Jackson R. Abernethy B. Wernhart S. (2009). Sensitivity to fine-grained and coarse visual information: the effect of blurring on anticipation skill. Int. J. Sport Psychol. 40, 461–475.

[B10] Laby D. M. Appelbaum L. G. (2021). Review: vision and on-field performance: a critical review of visual assessment and training studies with athletes. Optom. Vis. Sci. Off. Publ. Amer. Acad. Optom. 98, 723–731. 10.1097/OPX.0000000000001729 34328451

[B11] Langer M. S. Siciliano R. A. (2015). Are blur and disparity complementary cues to depth? Vis. Res. 107, 15–21. 10.1016/j.visres.2014.10.036 25482222

[B12] Limballe A. Kulpa R. Bennett S. (2022). Using blur for perceptual investigation and training in sport? A clear picture of the evidence and implications for future research. Front. Psychol. 12:752582. 10.3389/fpsyg.2021.752582 35308077 PMC8926072

[B13] Llanes-Jurado J. Marín-Morales J. Guixeres J. Alcañiz M. (2020). Development and calibration of an eye-tracking fixation identification algorithm for immersive virtual reality. Sensors 20:4956. 10.3390/s20174956 32883026 PMC7547381

[B14] Mann D. L. Abernethy B. Farrow D. (2010a). The resilience of natural interceptive actions to refractive blur. Hum. Mov. Sci. 29, 386–400. 10.1016/j.humov.2010.02.007 20430464

[B15] Mann D. L. Abernethy B. Farrow D. (2010b). Visual information underpinning skilled anticipation: the effect of blur on a coupled and uncoupled in situ anticipatory response. Atten. Percept. Psychophys. 72, 1317–1326. 10.3758/APP.72.5.1317 20601713

[B16] Miles H. C. Pop S. R. Watt S. J. Lawrence G. P. John N. W. (2012). A review of virtual environments for training in ball sports. Comput. Graph. 36, 714–726. 10.1016/j.cag.2012.04.00731724487

[B17] Negi S. Mitra R. (2020). Fixation duration and the learning process: an eye tracking study with subtitled videos. J. Eye Mov. Res. 13:10.16910/jemr.13.6.1. 10.16910/jemr.13.6.1 33828811 PMC8012014

[B18] Pannasch S. Velichkovsky B. M. (2009). Distractor effect and saccade amplitudes: further evidence on different modes of processing in free exploration of visual images. Vis. Cogn. 17, 1109–1131. 10.1080/13506280902764422

[B19] Petri K. Bandow N. Salb S. Witte K. (2019). The influence of facial expressions on attack recognition and response behaviour in karate kumite. Eur. J. Sport Sci. 19, 529–538. 10.1080/17461391.2018.1536170 30362894

[B20] Ripoll H. Kerlirzin Y. Stein J.-F. Reine B. (1995). Analysis of information processing, decision making, and visual strategies in complex problem solving sport situations. Hum. Mov. Sci. 14, 325–349. 10.1016/0167-9457(95)00019-O

[B21] Ryu D. Abernethy B. Mann D. L. Poolton J. M. (2015). The contributions of central and peripheral vision to expertise in basketball: how blur helps to provide a clearer picture. J. Exp. Psychol. Hum. Percept. Perform. 41, 167–185. 10.1037/a0038306 25485663

[B22] Ryu D. Abernethy B. Mann D. L. Poolton J. M. Gorman A. D. (2013). The role of central and peripheral vision in expert decision making. Perception 42, 591–607. 10.1068/p7487 24422243

[B23] Ryu D. Abernethy B. Park S. H. Mann D. L. (2018). The perception of deceptive information can be enhanced by training that removes superficial visual information. Front. Psychol. 9:1132. 10.3389/fpsyg.2018.01132 30174625 PMC6108161

[B24] Ryu D. Mann D. L. Abernethy B. Poolton J. M. (2016). Gaze-contingent training enhances perceptual skill acquisition. J. Vis. 16:2. 10.1167/16.2.2 26824639

[B25] Stone J. A. Strafford B. W. North J. S. Toner C. Davids K. (2018). Effectiveness and efficiency of virtual reality designs to enhance athlete development: an ecological dynamics perspective. Mov. Sport Sci. Sci. Motric. 4, 51–60. 10.1051/sm/2018031

[B26] van Biemen T. Koedijker J. Renden P. G. Mann D. L. (2018). The effect of blurred perceptual training on the decision making of skilled football referees. Front. Psychol. 9:1803. 10.3389/fpsyg.2018.01803 30319501 PMC6170623

[B27] Vater C. Williams A. M. Hossner E.-J. (2019). What do we see out of the corner of our eye? The role of visual pivots and gaze anchors in sport. Int. Rev. Sport Exerc. Psychol. 13, 81–103. 10.1080/1750984X.2019.1582082

[B28] Williams A. Jackson R. (2019). Anticipation in sport: fifty years on, what have we learned and what research still needs to be undertaken? Psychol. Sport Exerc. 42, 16–24. 10.1016/j.psychsport.2018.11.014

[B29] Williams A. M. Elliott D. (1999). Anxiety, expertise, and visual search strategy in karate. J. Sport Exer. Psychol. 21, 362–375. 10.1123/jsep.21.4.362

